# MRI-only based material mass density and relative stopping power estimation via deep learning for proton therapy: a preliminary study

**DOI:** 10.1038/s41598-024-61869-8

**Published:** 2024-05-15

**Authors:** Yuan Gao, Chih-Wei Chang, Sagar Mandava, Raanan Marants, Jessica E. Scholey, Matthew Goette, Yang Lei, Hui Mao, Jeffrey D. Bradley, Tian Liu, Jun Zhou, Atchar Sudhyadhom, Xiaofeng Yang

**Affiliations:** 1 Department of Radiation Oncology and Winship Cancer Institute, Emory University, Atlanta, GA 30308 USA; 2 GE Healthcare, Atlanta, GA 30308 USA; 3 Department of Radiation Oncology, Dana-Farber Cancer Institute, Brigham and Women’s Hospital, and Harvard Medical School, Boston, Massachusetts USA; 4 Department of Radiation Oncology, The University of California, San Francisco, CA 94143 USA; 5 Department of Radiology and Imaging Sciences and Winship Cancer Institute, Emory University, Atlanta, GA USA; 6 Radiation Oncology, Mount Sinai Medical Center, New York, NY USA

**Keywords:** Cancer imaging, Radiotherapy

## Abstract

Magnetic Resonance Imaging (MRI) is increasingly being used in treatment planning due to its superior soft tissue contrast, which is useful for tumor and soft tissue delineation compared to computed tomography (CT). However, MRI cannot directly provide mass density or relative stopping power (RSP) maps, which are required for calculating proton radiotherapy doses. Therefore, the integration of artificial intelligence (AI) into MRI-based treatment planning to estimate mass density and RSP directly from MRI has generated significant interest. A deep learning (DL) based framework was developed to establish a voxel-wise correlation between MR images and mass density as well as RSP. To facilitate the study, five tissue substitute phantoms were created, representing different tissues such as skin, muscle, adipose tissue, 45% hydroxyapatite (HA), and spongiosa bone. The composition of these phantoms was based on information from ICRP reports. Additionally, two animal tissue phantoms, simulating pig brain and liver, were prepared for DL training purposes. The phantom study involved the development of two DL models. The first model utilized clinical T1 and T2 MRI scans as input, while the second model incorporated zero echo time (ZTE) MRI scans. In the patient application study, two more DL models were trained: one using T1 and T2 MRI scans as input, and another model incorporating synthetic dual-energy computed tomography (sDECT) images to provide accurate bone tissue information. The DECT empirical model was used as a reference to evaluate the proposed models in both phantom and patient application studies. The DECT empirical model was selected as the reference for evaluating the proposed models in both phantom and patient application studies. In the phantom study, the DL model based on T1, and T2 MRI scans demonstrated higher accuracy in estimating mass density and RSP for skin, muscle, adipose tissue, brain, and liver. The mean absolute percentage errors (MAPE) were 0.42%, 0.14%, 0.19%, 0.78%, and 0.26% for mass density, and 0.30%, 0.11%, 0.16%, 0.61%, and 0.23% for RSP, respectively. The DL model incorporating ZTE MRI further improved the accuracy of mass density and RSP estimation for 45% HA and spongiosa bone, with MAPE values of 0.23% and 0.09% for mass density, and 0.19% and 0.07% for RSP, respectively. These results demonstrate the feasibility of using an MRI-only approach combined with DL methods for mass density and RSP estimation in proton therapy treatment planning. By employing this approach, it is possible to obtain the necessary information for proton radiotherapy directly from MRI scans, eliminating the need for additional imaging modalities.

## Introduction

Proton radiotherapy has an advantage over photon radiation therapy because of the proton beam Bragg peak effect^1,2^. Protons stop in tissue immediately after depositing most of their energy, sparing surrounding normal tissue. In practice, proton treatment planning systems (TPSs) calculate dose deposited by a proton beam using three-dimensional (3D) maps of relative stopping power (RSP) or mass density. Currently, patient-specific maps of RSP acquired with volumetric imaging are used to translate CT number acquired using single-energy computed tomography (SECT) with appropriate conversions and calibration coefficients via stoichiometric calibration^3,4^. The accuracy of this approach relies on chemical composition difference between patient tissue and tissue substitute phantoms used in calibration^5,6^. Research has shown that the tissue substitute used for stoichiometric calibration can be an additional uncertainty in proton range estimation^7^. Since SECT cannot distinguish differences in CT number as a result of changes in mass density or material chemical composition^8^, the error in mass density and RSP calculation can become relevant. CT image noise and artifacts also contribute to the error in mass density and RSP calculation^9^. To account for these uncertainties, additional margins of 2.5%-3.5% of the proton beam range are added in clinical practice^10,11^.

Magnetic Resonance Imaging (MRI) is increasingly incorporated into treatment planning because of its superior soft tissue contrast used for tumor and soft tissue delineation versus CT. Importantly, MRI can reduce inter- and intra-observer contouring variability in many disease sites^12,13^. In most current workflows, target and organ at risk (OAR) contours defined from MRI are transferred to CT via image registration. This approach introduces geometrical uncertainties of 2–3 mm depending on disease site^14,15^, which are systematic errors and could lead to a geometric miss that compromises tumor control in proton radiotherapy^16^. Thus, MRI-only treatment planning studies for radiotherapy have been performed in recent decades which could increase geometric treatment accuracy by removing CT-to-MRI registration uncertainty^17–19^, and a reduction in patient received radiation, time, and cost.

However, unlike CT, MRI cannot provide mass density or RSP maps required for proton radiotherapy dose calculation. Several approaches have been proposed to overcome this limitation by generating CT-like images to derive electron density maps, including atlas-based electron density mapping^20,21^ and synthetic CT-based methods^22–24^. Sudhyadhom et al*.* presented a method to determine material mean ionization potential from MRI^25,26^. Based on these methods for estimating material physical properties from MRI, Scholey et al*.* recently proposed a combined CT-MRI-based workflow for estimating RSP, which has demonstrated RSP uncertainty within 1% for soft tissue^27^. That research inspired us to explore the feasibility of correlating mass density and RSP information using only MRI.

Interest in incorporating artificial intelligence (AI) into treatment planning has risen in recent years. Su et al*.* demonstrated that artificial neural networks (ANN) outperformed the traditional machine learning method based on their phantom experiment using dual-energy CT (DECT)^28^. Meanwhile, deep learning (DL) featured in hierarchical structures has been deployed to solve multi-physics inverse problems^29,30^. Numerous research efforts have presented on the feasibility of incorporating DL into mass density or RSP estimation for proton therapy based on SECT^31,32^, DECT^33–35^, or cone beam CT (CBCT)^36^. Liu et al*.*’s work^37^ has shown the potential of using deep learning (DL) methods to generate synthetic DECT images from MRI scans, and these synthetic DECT images can be utilized for treatment planning. However, this approach still hinges on the accuracy of the synthetic DECT, the calibration of the CT curve, and the errors involved in registering MRI with DECT. In our study, we aim to bypass these limitations by establishing a direct relationship between MRI data and both mass density and RSP. Our approach is particularly intriguing as it integrates DL networks into MRI-only treatment planning, developing a model that translates MRI data directly into mass density or RSP values. This direct translation is what distinguishes our work from previous studies. We propose a novel framework that employs DL to accurately estimate material mass density and RSP specifically for proton radiotherapy, based solely on MRI. This direct approach could streamline the treatment planning process, reducing dependencies on additional imaging modalities and mitigating associated errors.

## Materials and methods

### Tissue substitute phantoms and data collection

Five tissue substitute phantoms and two animal tissue phantoms were included in this experiment. Phantom dimensions were 5.7 × 5.7 × 12.9 cm^3^. Phantoms were placed into a cylindrical water container (8.41 cm radius and 25.4 cm height) and secured with tape and Styrofoam for stability. The dimensions of the cylindrical water container is very close to adult human body’s head-and-neck^38^. The water container was filled with deionized water. The five tissue substitute phantoms were created to mimic skin, muscle, adipose, spongiosa, and 45% HA bone, based on each tissue’s molecular composition provided in ICRU Report 44 and ICRP Report 23, and further modified by Scholey et al.^5,27,39^. Cortical bone, which lacks free water molecules, cannot be detected by MRI. Therefore, we simulated its density with a different composition, and named this phantom "45% HA bone." The tissue substitute phantoms were made from homogeneous mixtures of deionized water with specific ratios of porcine skin gelatin (protein substitute), porcine lard (fat substitute), hydroxyapatite (bone substitute) and a small amount of detergent/surfactant (SDS) to enhance homogeneity. Detailed material information is shown in Table 1. The two animal tissue phantoms were made from pig blood mixed with either finely minced pig brain or liver. In our study, we took precautions to maintain the freshness and integrity of the animal tissue phantoms used. These phantoms were stored in a refrigerator, and all measurements were performed within a week to ensure that the material remained fresh during the experiments. The mass density of each animal tissue phantom was measured using a high precision scale (Practum313-1S, Sartorius Biotech, Germany) and volumetric pipettes. The chemical composition of each phantom was measured by combustion analysis at a specialized microanalytical facility. The mean excitation energy of each phantom was calculated using the Bragg additivity rule^40^. RSP of the tissue substitute and animal tissue phantoms was measured with Varian ProBeam System (Varian Medical Systems, Palo Alto) and Zebra (IBA Dosimetry, Germany), as described by Chang et al.^41^. The proton spot was set at 150 MeV with a cyclotron current of 50 nA. The water equivalent thickness (WET) was calculated by taking the difference between the empty container and each phantom. RSP was calculated by dividing the WET with container width. The measured material information for tissue substitute and animal tissue phantoms are shown in Table 2**.**

**Table 1:** Mass percent compositions for each tissue substitute phantom.

Tissue substitute phantoms	Water	Gelatin (protein)	Lard (fat)	Hydroxyapatite	SDS
Skin	75.0	25.0			
Muscle	74.78	19.97	5.0		0.25
Adipose			100		
Spongiosa	26.61	11.83	47.43	12.81	1.32
45% HA bone	55.0			45.0

**Table 2:** Measured mass density (*ρ*_*meas*_), RSP, mean excitation energies (*I*), and elemental mass percent compositions for each tissue substitute and animal tissue phantom.

Tissue substitute and animal tissue phantoms	*ρ*_*meas*_ (g/cm^3^)	*RSP*	*I* (eV)	H	C	N	O	Na	P	S	Ca
Skin	1.077	1.067	77.5	10.09	10.55	3.82	75.26			0.28	
Muscle	1.064	1.056	76.7	10.37	12.44	3.06	73.86	0.02		0.25	
Adipose	0.936	0.979	61.9	12.37	77.70	0.16	9.77				
Spongiosa	1.091	1.076	76.1	9.79	42.50	1.88	37.96	0.11	2.37	0.28	5.11
45% HA bone	1.417	1.344	108.5	6.24			67.48		8.32		17.96
Brain	1.028	1.014	77.3	10.96	6.02	0.73	82.24			0.05	
Liver	1.065	1.054	77.2	10.60	8.98	2.05	78.25			0.12	

**Figure 1:**
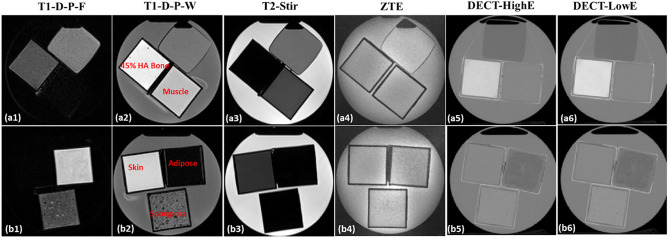
Axial images of tissue substitutes acquired using different MRI and DECT sequences: (**a1**–**a6**) represent T1-weighted Dixon-predicted water-only (T1-D-P-W), T1-weighted Dixon-predicted fat-only (T1-D-P-F), T2-weighted short tau inversion recovery (T2-STIR), zero echo time sequence (ZTE), DECT high-energy (DECT-HighE), and DECT low-energy (DECT-LowE) images, respectively (**b1**–**b6**) are the same order of different MRI and DECT scans for another phantom cylinder.

**Figure 2:**
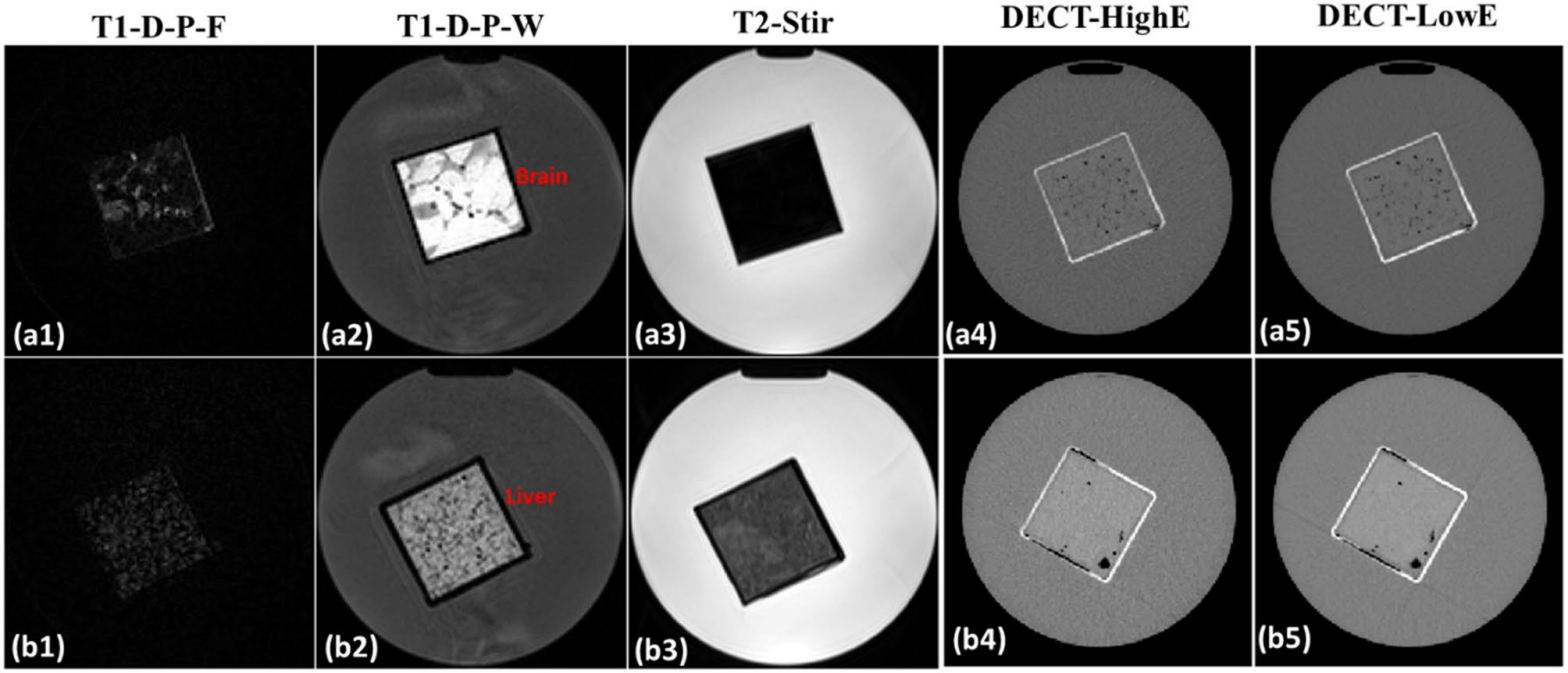
Axial images of tissue substitutes (brain) acquired using different MRI and DECT sequences: (**a1**–**a5**) represent T1-weighted Dixon-predicted water-only (T1-D-P-W), T1-weighted Dixon-predicted fat-only (T1-D-P-F), T2-weighted short tau inversion recovery (T2-STIR), DECT high-energy (DECT-HighE), and DECT low-energy (DECT-LowE) images, respectively (**b1**–**b5**) are the same order of different MRI and DECT scans for another animal phantom (liver) cylinder.

The five tissue substitute phantoms were scanned using a 1.5 T Siemens MAGNETOM Area MRI scanner using a T1-weighted Dixon VIBE sequence, used to generate T1-weighted Dixon VIBE predicted fat (T1-D-P-F) and T1-weighted Dixon VIBE predicted water (T1-D-P-W) images, and a T2-weighted short tau inversion recovery Sampling Perfection with Application optimized Contrasts using different flip angle Evolution (SPACE) sequence for T2-STIR images. To maximize signal in bony tissues, the tissue substitute phantoms were scanned using a 3.0 T GE SIGNA PET/MR using a zero echo time (ZTE) sequence^27^ to compare with the T1 and T2 MRIs. The two animal tissue phantoms were also scanned using the 1.5 T Siemens MAGNETOM Area MRI scanner to acquire T1 Dixon VIBE and T2-STIR sequences. T1 and T2 MRI matrices were 208 × 288 × 176 and 256 × 256 × 208, while ZTE MRI matrix was 256 × 256 × 60. MRI acquisition parameters are shown in Table 3. The MR voxel size, as well as sequence parameters like TR and TE, were selected to align with the routine treatment simulation protocol. We conducted a thorough assessment of the signal-to-noise ratio (SNR) for each MR image across various sequences. Notably, even in the sequence with the lowest SNR, the value remained above 30, which is well above the minimum threshold for adequate image quality in 3D gradient echo imaging. This suggests that any potential degradation in image quality can be safely disregarded, as the contrast observed in each voxel can be reliably attributed to the tissue properties. The obtained T1-D-P-W, T1-D-P-F, T2-STIR, ZTE MR phantom images are shown in Figs. 1 and Fig. 2.

**Table 3:** MRI T1, T2 and ZTE acquisition parameters.

Sequence	Scanner	3D voxel size (mm^3^)	Repetition time-TR (ms)	Echo time-TE (ms)
T1	1.5 T Siemens MAGNETOM Area	1.247 × 1.247 × 1.2	7.76	2.39
T2	1.5 T Siemens MAGNETOM Area	1.016 × 1.016 × 1.1	3500	248
ZTE	3.0 T GE SIGNA PET/MR	0.8 × 0.8 × 1	700.7	0

Tissue substitute and animal tissue phantoms were also scanned with a Siemens SOMATOM Definition Edge CT scanner using a head-and-neck (HN) TwinBeam dual-energy (TBDE) protocols with a CT dose index (CTDI_vol, 32 cm_) of 8.6 mGy and effective radiation produced of 400 mAs_eff_. In this work, DECT images acquired at 120 kVp with gold (Au) and tin (Sn) filters were referenced as DECT high-energy (HighE) and low energy (LowE), respectively. Detailed information for DECT scans is shown in Table 4. The FOV is bigger than the phantom, since we want to follow the clinical protocol to reconstruct the CT images such that the results are consistent with patient conditions. The phantoms were remained in the water cylinder (8.41 cm radius and 25.4 cm height) to emulate a head phantom. All DECT images were reconstructed using the same settings with a diameter of 500 mm and slice thickness of 0.5 mm. Maps of relative electron density (*ρ*_*e*_) and effective atomic number (Z_eff_) were generated using Siemens Syngo.Via to calculate mass density and RSP as reference. Siemens Syngo.Via generated the parametric maps using DECT material decomposition, which have mean errors of 0.8% (${\rho }_{e}$) and 2.9% (Z_eff_)^42^, respectively. Syngo.via method for parametric map generation has been validated by research^43,44^. The mass density and RSP are calculated by DECT empirical functions shown in Eqs. (1) and (2). The phantom MRI images were first rigidly registered to DECT images, and then deformable image registration was applied using Velocity™. Then, the MRI images were resampled to match the resolution of DECT images.

**Table 4:** DECT acquisition parameters.

Scanner	Siemens SOMATOM definition edge
Collimation	64 × 0.6 mm
Voxel size	0.977 × 0.977 × 0.5 mm^3^
Field of view	500 mm
X-ray tube voltage	120 kVp with Au and Sn filters
Reconstruction method	Sinogram-affirmed iterative reconstruction (SAFIRE)
Beam hardening correction	Qr40 iterative beam hardening correction (Syngo.CT VB20A)

### MRI based mass density and RSP mapping using deep learning framework

#### Phantom study

**Figure 3:**
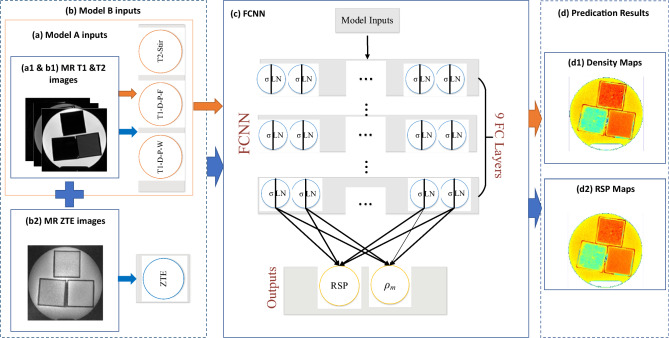
MRI-based mass density and RSP estimation with deep learning method framework for phantom study, (**a**) Model A contains MRI T1&T2 images as input, (a1 and b1) MRI T1-D-P-W,T1-D-P-F and T2-Stir images for Model A and Model B, (b2) MRI ZTE images, (**b**) Model B have MRI T1,T2 and ZTE images as inputs, (**c**) FCNN network structure, (**d**) predication results, (d1) phantom mass Density maps, (d2) RSP maps. Orange arrows show Model A inputs and outputs while blue arrows show Model B inputs and outputs. The symbols σ and LN represent the activation functions used, specifically ReLU (Rectified Linear Unit) and layer normalization. Each fully connected layer in our model consists of 80 nodes.

**Figure 4:**
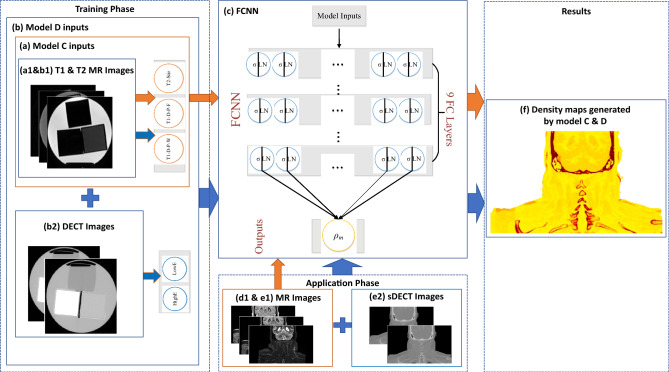
MRI-based mass density with deep learning method framework for patient application, (**a**) Model C has phantom MRI T1 and T2 images as input, (**b**) Model D has phantom MRI T1,T2 and DECT images, (a1 and b1) phantom MRI T1 and T2 images for model C and model D, (b2) phantom DECT images, (**c**) FCNN network, (d1 and e1) patient MRI T1 and T2 images, (e2) patient synthetic DECT images generated from (d1 and e1), (**f**) patient mass density maps. Orange arrows show Model C inputs and outputs for training phase and application phase, blue arrows show Model D inputs and outputs for training phase and application phase. The symbols σ and LN represent the activation functions used, specifically ReLU (Rectified Linear Unit) and layer normalization. Each fully connected layer in our model consists of 80 nodes.

Both phantom and patient application frameworks were investigated for this study, as shown in Figs. 3 and 4. In the phantom study, two deep learning models with different inputs were investigated. The inputs of model A includes MR T1-D-P-F, T1-D-P-W and T2-STIR images. Model A was trained with five substitute tissue and two animal tissue phantoms. Model B was trained with five tissue substitute phantoms’ MR T1-D-P-F, T1-D-P-W, T2-STIR and ZTE images. Mass density and RSP values estimated using Model A and B were compared in five tissue substitutes. Mass density and RSP values estimated using Model A, Model B, and DECT-based reference models were compared in five tissue substitute and two animal tissue phantoms. Model A was trained with clinical MRI modality and was developed to demonstrate the feasibility of MRI only treatment planning. Model B incorporated an additional MRI sequence, ZTE, and was developed to evaluate its advantage on bone tissue. In the phantom study, a stringent separation strategy was meticulously followed for the training, validation, and application datasets. This involved the use of a "half and half" approach, where the first half of the MRI slices served as the exclusive training dataset, and the second half was dedicated to the application dataset. In the training dataset, 75% of the pixel values were randomly chosen from the training dataset for model training, while the remaining 25% of pixel values were allocated for validation purposes. This method was designed to ensure that there was absolutely no overlap between these two datasets, guaranteeing that the model was trained and evaluated on entirely distinct sets of data. Such segregation is essential for evaluating how effectively the model generalizes to new and unseen data, a critical factor for its real-world applicability.

#### Patient application study

Figure 4 shows the MRI-based framework for patient mass density estimation. In this part, two deep learning models (C and D) were trained with tissue phantoms’ MRI and DECT images to predict mass density. Patient MRI and DECT images were registered using rigid and deformable methods. In the training phase, our patient dataset did not include MR T1-D-P-F images, so we developed Model C, which includes all 7 phantoms (5 tissue substitute phantoms and two animal tissue phantoms) T1-D-P-W and T2-STIR MR images. To demonstrate the feasibility of sDECT on the treatment planning, Model D was developed. In the training phase, Model D was trained with all 7 phantoms' T1-D-P-W and T2-STIR MRIs, DECT HighE and DECT LowE images. In the application phase, the inputs of Model C were patient T1-D-P-W and T1-D-P-F MRIs. Model D includes patient T1-D-P-W, T2-STIR and sDECT (Synthetic DECT) HighE, LowE MRIs as inputs. The patient T1-D-P-W, T1-D-P-F MRIs were obtained from Velocity™.sDECT images including sDECT HighE and LowE images were generated from MRI T1-D-P-W images using a deep learning method^37^.

Each patient underwent a two-step imaging process for proton treatment planning. Initially, a CT simulation was performed on a Siemens SOMATOM Definition Edge Twin-beam CT scanner, operating in DECT (Dual-Energy CT) mode. Subsequently, an MRI scan was conducted on a Siemens Aera 1.5 T scanner, all on the same day. Both the DECT and MRI images were utilized in the treatment planning process. The MRIs were acquired in the form of 3D T1-weighted maps with the following parameters: TE (Echo Time) / TR (Repetition Time) = 2.4/7.8 ms, Flip angle = 12°, Pixel spacing of 1.2 × 1.2 × 1.2 mm^3^, Each slice had dimensions of 288 × 192 pixels. For each patient, the MRI data underwent a deformable registration process and was resampled to align with the DECT data. This registration and resampling were performed using commercial medical image processing software that has received approval for clinical use, namely Velocity AI 3.2 by Varian Medical Systems, based in Palo Alto, CA. This registration and resampling step ensures that the MRI data can be effectively integrated into the treatment planning process alongside the DECT data, the accuracy of deformable image registration is discussed in this publication^45^, the detail information can be found in previous study^37^. Of note, there is no additional dose applied to patients in this study, since synthetic DECT is estimated from MRI. The four deep learning models based on MRI and sDECT images for phantom and patient studies are shown in Table 5.

**Table 5:** Deep learning models for phantom study and patient application, the image modalities of inputs, training targets, training outputs are shown.

Training set	Deep learning models	Inputs	Outputs
Substitute tissue and animal tissue phantoms	Model A	T1-D-P-W, T1-D-P-F, T2-STIR	Phantom mass density map, Phantom RSP map
Substitute tissue phantoms	Model B	T1-D-P-W, T1-D-P-F, T2-STIR, ZTE	Phantom mass density map, Phantom RSP map
Substitute tissue and animal tissue phantoms	Model C	T1-D-P-W, T1-D-P-F	Patient mass density map
Substitute tissue and animal tissue phantoms	Model D	T1-D-P-W, T1-D-P-F, sDECT 120 kVp Au filter (HighE), sDECT 120 kVp Sn filter (LowE),	Patient mass density map

### Deep learning model and training data preparation

In a previous study, we conducted a comparison of three deep learning models to estimate mass density and RSP (Relative Stopping Power) using single-energy CT (SECT) images. The results indicated that fully connected neural networks (FCNN) exhibited superior performance and lower computational costs^31^. In this study, a 1-dimension (1D) FCNN^46^ was built on Pytorch^47^ to perform mass density and RSP estimation based on MR images. The details of the FCNN model are shown in Fig. 3c and for more comprehensive details, please refer to our previous studies^31,34,41^. Round volumes of interest (VOI) were manually contoured for 7 tissue phantoms with a radius of 15 voxels for MRI and DECT images. In the phantom study, the first half of MRI and DECT images slices were chosen as the training dataset, while the remaining slices were chosen as the application dataset. Phantom MRI and DECT images were registered using rigid and deformable method. The number of extracted voxels in each tissue phantom of each slice is 24,000. The total number of voxels in the training data was 168,000 for Model A and 120,000 for Model B (Animal tissue phantoms were not scanned with MRI ZTE). The DL models inputs consisted of signals from T1, T2, and ZTE MRIs at each voxel, which were organized into 1D arrays and combined to form input matrices with dimensions of 168,000 × 3 (for Model A) and 120,000 × 4 (for Model B). In the case of Model A, there are three modalities as inputs, resulting in a total of 3 values per voxel (T1-D-P-W,T2-D-P-F,T2-STIR). For Model B, which incorporates four modalities, there are 4 different values associated with each specific voxel (T1-D-P-W, T2-D-P-F,T2-STIR,ZTE). Here, the numbers 168,000 and 120,000 represent the total number of voxels in the input dataset, and the "×3" and "×4" indicate that at each specific voxel, there are 3 or 4 different values corresponding to the MR modalities. In the patient application study, all slices of tissue phantoms were input to the training dataset, meaning each input had 336,000 voxels. The input matrix from patient images input to the model has a size of 336,000 × 2 (Model C) and 336,000 × 4 (Model D) since Model C and Model D have 2 and 4 modality images as inputs. The loss function employed to supervise the DL models was defined as the discrepancy between the true and predicted values at each voxel, as expressed in Eqs. (1) and (2).Where ${\rho }_{i,DL}$ and ${RSP}_{i,DL}$ denote the DL model predicted mass density and RSP at specific voxel i, while ${\rho }_{i,grd\_truth}$ and ${RSP}_{i,grd\_truth}$ denote the ground mass density and RSP at specific voxel i. The operator ${\Vert \Vert }_{2}^{2}$ signifies the mean squared error (MSE) calculator.

1 $${\mathcal{L}}_{\rho }=\frac{1}{N}\sum_{i=1}^{N}{\Vert {\rho }_{i,DL}-{\rho }_{i,grd\_truth}\Vert }_{2}^{2}$$

2 $${\mathcal{L}}_{RSP}=\frac{1}{N}\sum_{i=1}^{N}{\Vert {RSP}_{i,DL}-{RSP}_{i,grd\_truth}\Vert }_{2}^{2}$$

The variation in MR pixel values is often a consequence of disparities in scanning sequences and equipment configurations. Nonetheless, it is crucial to maintain consistent contrast values for effective differentiation among different scans. To address this challenge, we have introduced a robust normalization procedure for MR pixel values in our methodology. In this normalization process, we establish a standardized reference point for each scan by computing the average pixel values associated with pure water within that specific dataset. By utilizing the average pixel value of water as the reference value, we ensure that the contrast values are consistently calibrated, irrespective of the inherent differences stemming from diverse MR imaging conditions. This normalization technique not only mitigates the impact of sequence and equipment variations but also enhances the reliability and comparability of our results.

The optimization process involved varying the number of fully connected (FC) layers within the range of 5 to 11, and it was determined that the configuration with 9 FC layers yielded the best performance during the optimization tests. Each of these FC layers consisted of 80 nodes. All the neural networks were trained using an NVIDIA GeForce 4090 GPU equipped with 24 GB of memory, and a batch size of 1000 was employed. And each model was trained with 500 epochs. The learning rate for the Adam optimizer was set to 1e-3. During the training process, each batch optimization operation consumed approximately 0.5 GB of CPU memory and 2 GB of GPU memory. It's important to note that all the networks were implemented in PyTorch version 2.1.

### Empirical model based on DECT parametric mapping and linear regression method.

To compare DL models for estimating mass density and RSP based on MRI images, an empirical model based on DECT parametric mapping was implemented in this work. The substitute tissue and animal tissue phantoms were scanned with a Siemens SOMATOM Definition Edge CT scanner; scanner set up and data collection information is shown in Table 4. The DECT parametric maps were obtained from Siemens Syngo.Via. Equation (3) ^48^ and Eq. (4) ^49,50^ give empirical models to which can be used to estimate the material mass density and RSP maps from DECT, where ${\rho }_{e}$ and ${Z}_{eff}$ are obtained from Syngo.Via. In the phantom study, the mass density and RSP models were applied to the application dataset and compared with DL models (Models A & B) based on MR images. In the patient application study, the mass density model was applied on patient DECT images and compared with DL models (Model C&D) based on MR and sDECT images.

3 $$\rho =-0.1746+1.176{\rho }_{e}$$

4 $$RSP=\left\{\begin{array}{c}\begin{array}{c}{\rho }_{e,} \\ \left(1.1114-0.0148{Z}_{eff}\right){\rho }_{e}\\ 0.9905{\rho }_{e,} \end{array}\\ \left(1.1117-0.0116{Z}_{eff}\right){\rho }_{e,}\end{array}\right.\begin{array}{c} 0\le {Z}_{eff}<0.5\\ \begin{array}{c}0.5\le {Z}_{eff}<8.5\\ \begin{array}{c}8.5\le {Z}_{eff}<10\\ {Z}_{eff}\ge 10\end{array}\end{array}\end{array}$$

Deep learning (DL) is frequently viewed as a black-box technique because of its complex nature. To offer a more thorough assessment of our approach, we have incorporated a simple linear regression model for comparative analysis. This model differs from other DL models, as it specifically utilizes the T1-D-P-W dataset, in contrast to other DL models that are based on the T1 and T2 datasets. The linear regression performed with MATLAB.

### Evaluation method

The ground truth data was generated with the reference mass density and RSP of each phantom shown in Table 2. Absolute percentage error (APE) and mean absolute percentage error (MAPE) shown in Eqs. (5) and (6) were used to evaluate the performance of DL models based on MR images over each phantom, where *i* denotes the *i*th voxel, x is the mass density or RSP at specific voxel, and N is the total voxels. In the phantom study, tables of MAPE for each tissue phantom were made to show the performance of each model. The MAPE metrics was applied to the application dataset.

5 $${APE}_{i}=\left|\frac{{x}_{i}-{x}_{i,REF}}{{x}_{i,REF}}\right|\times 100\%$$

6 $$MAPE=\frac{1}{N}\sum_{i=1}^{N}{APE}_{i}$$

### Ethical statement

The ethics committee, Emory Institutional Review Board (Emory IRB), had reviewed and approved this study (IRB #114349), and informed consent was not required by Emory IRB for this Health Insurance Portability and Accountability Act (HIPAA) compliant retrospective analysis. All methods were carried out in accordance with relevant guidelines and regulations.

## Results

MAPE was used to evaluate the performance of DL models (Models A and B) based on MR images for estimating mass density and RSP for each phantom. In the patient application study, mass maps and line profiles at specific tissues were made to visualize the comparison between models (Models C and D).

### Tissue substitute and animal tissue phantoms: mass density and RSP estimation analysis

Table 6 shows the MAPE comparisons of mass densities between MRI-based models and the DECT empirical model. The results indicate that linear regression performed less effectively across all tissue phantoms. In contrast, DL models based on MR images (Models A and B) demonstrated better performance than the DECT empirical model over all tissue substitute and animal tissue phantoms. The DL models based on MR images also have smaller standard deviation than the DECT empirical model, except for brain. Model A has conventional T1 and T2 MR images as input, and it demonstrated the best performance in skin, muscle, adipose, brain, and liver. In addition to the T1 and T2 MR images, inputs of Model B included ZTE MR images, and it demonstrated the best performance in spongiosa and 45% HA bone. Table 7 shows the MAPE comparison of RSP between MRI-based models and the DECT empirical model. The results indicate that linear regression performed less effectively across all tissue phantoms. In contrast, MRI-based DL models (Models A and B) demonstrated better performance than the DECT empirical model in all seven phantoms. The MRI-based DL models have smaller standard deviation than the DECT empirical model, except for brain. Model A includes conventional T1 and T2 MR images as input and demonstrated lower RSP MAPE in skin, muscle, adipose, brain and liver. The ZTE MR images were added as an extra input to Model B, which demonstrated better performance than Model A and the DECT empirical model in spongiosa and 45% HA bone. The DL models exhibited lower prediction accuracy on the animal tissue phantom compared to the tissue substitute phantom. This discrepancy can be attributed to the differences in the homogeneity levels between the two types of phantoms. The tissue substitute phantom was meticulously composed of water, gelatin, lard, hydroxyapatite powder, and SDS, allowing for thorough mixing and achieving a high level of homogeneity that the animal tissue phantom couldn't attain. This distinction in homogeneity is evident when examining Fig. 2.

**Table 6:** MAPE comparison of mass densities between two MRI based FCNN models and DECT empirical model, and linear regression model.

Phantom	Linear regression (T1)	DECT empirical	FCNN MRI based (T1 and T2) (Model A)	FCNN MRI based (T1, T2, ZTE) (Model B)
Skin	19.83 ± 8.65	0.77 ± 0.53	**0.42 ± 0.21**	0.44 ± 0.03
Muscle	29.35 ± 13.81	0.69 ± 0.52	**0.14 ± 0.40**	0.15 ± 0.02
Adipose	17.34 ± 1.97	4.61 ± 3.38	**0.19 ± 0.11**	0.26 ± 0.07
Spongiosa	27.07 ± 3.51	1.21 ± 1.13	0.33 ± 0.32	**0.23 ± 0.61**
45% HA Bone	7.04 ± 20.52	1.47 ± 3.00	0.82 ± 2.73	**0.09 ± 0.70**
Brain	18.62 ± 17.37	1.73 ± 2.50	**0.78 ± 3.47**	N/A
Liver	7.32 ± 12.37	0.94 ± 1.74	**0.26 ± 0.27**	N/A

Significant values are in bold.

**Table 7:** MAPE comparison of RSP between linear regression model, DECT empirical model and two MRI based FCNN models.

Phantom	Linear regression (T1)	DECT empirical	FCNN MRI based (T1 and T2) (Model A)	FCNN MRI based (T1, T2, ZTE) (Model B)
Skin	22.29 ± 7.78	0.57 ± 0.43	**0.30 ± 0.16**	0.34 ± 0.03
Muscle	26.51 ± 12.93	0.58 ± 0.42	**0.11 ± 0.04**	0.13 ± 0.02
Adipose	28.57 ± 1.91	4.34 ± 3.28	**0.16 ± 0.08**	0.24 ± 0.05
Spongiosa	31.05 ± 3.30	1.17 ± 1.23	0.29 ± 0.31	**0.19 ± 0.43**
45% HA Bone	1.98 ± 22.51	1.32 ± 2.76	0.67 ± 2.13	**0.07 ± 0.55**
Brain	16.62 ± 17.67	1.43 ± 2.40	**0.61 ± 2.57**	N/A
Liver	9.32 ± 10.28	0.84 ± 1.76	**0.23 ± 0.19**	N/A

Significant values are in bold.

### Head-and-neck patient application study

**Figure 5:**
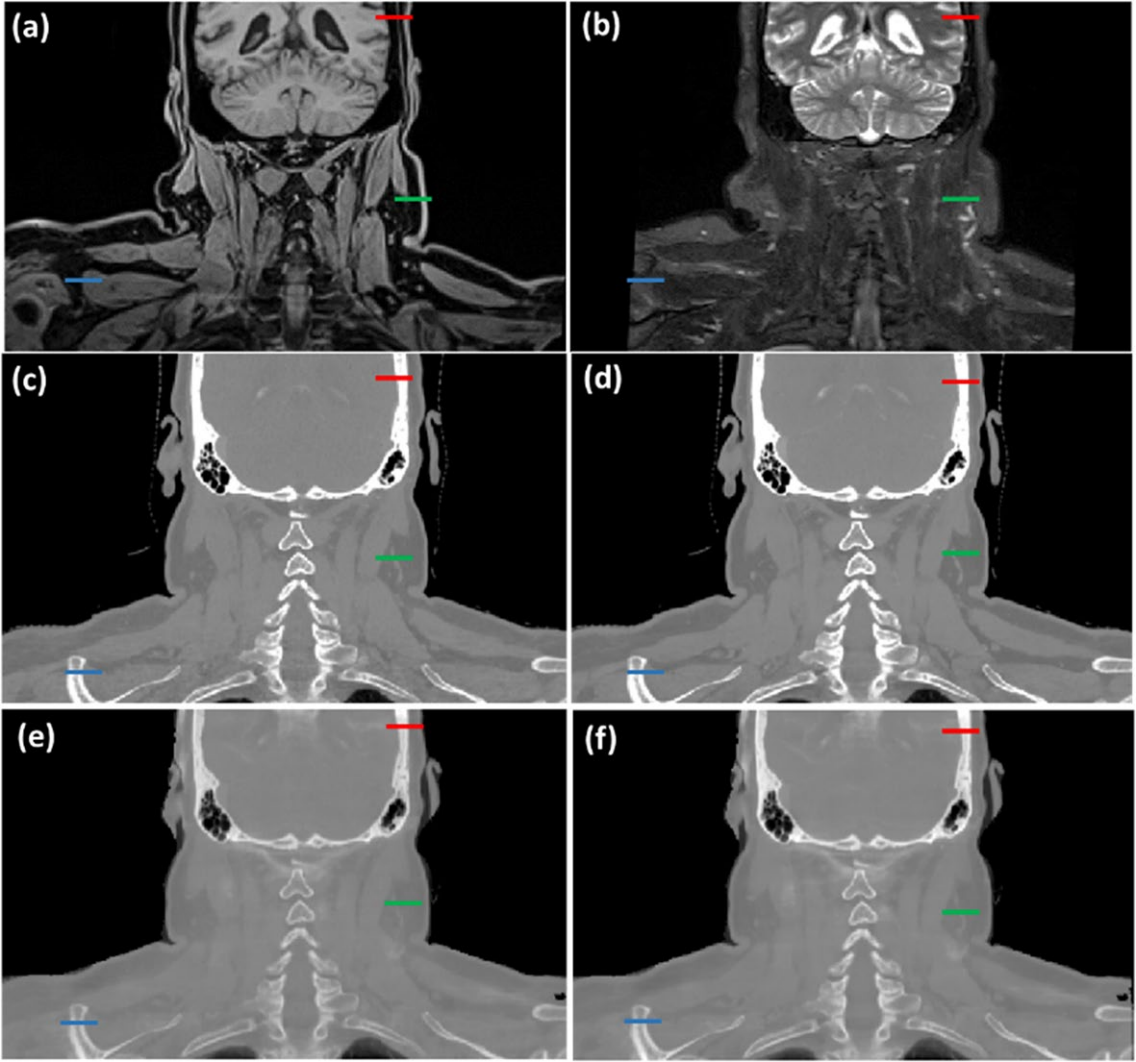
Patient coronal images from, (**a**) T1-weighted Dixon predicated water-only (T1-D-P-W), (**b**) T2-weighted short tau inversion recovery (T2-stir), (**c**) DECT high-energy, (**d**) DECT low-energy, (**e**) sDECT high-energy, (**f**) sDECT low-energy. (**a**,**b**) are MR scans image from the patients, (**c**,**d**) are the DECT scans for that patient, (**e**,**f**) are the synthetic DECT images generated based on (**a**,**b**) using established method describe in our previous work^37^. The red, yellow, and green bars indicate specific locations used for line profile comparison, as demonstrated in the results presented in Fig. 6. The profile comparisons are shown in Fig. 7.

**Figure 6:**
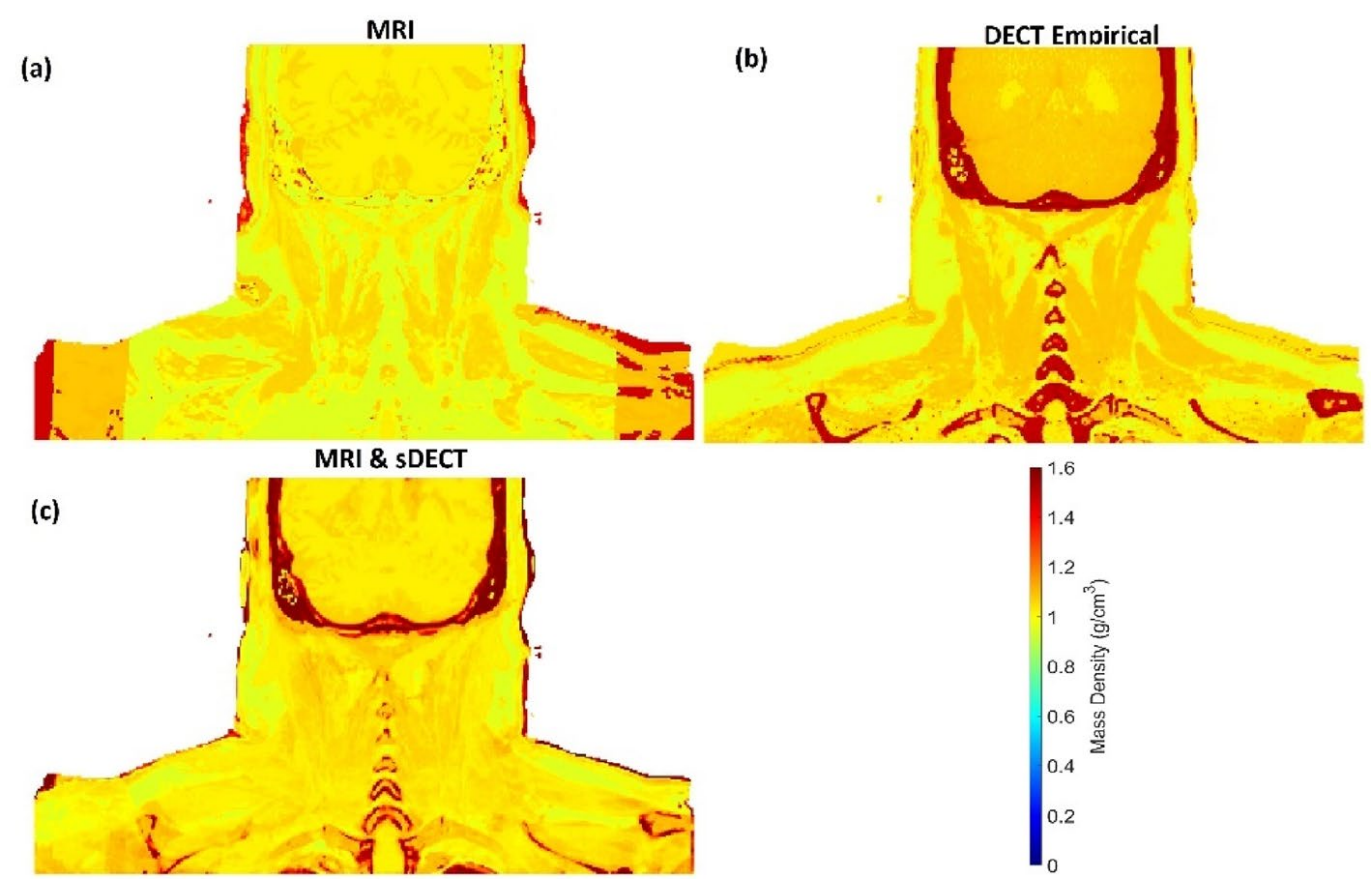
Patient mass density maps are generated using different methods, including (**a**) T1 and T2 MRI-based (Model C), (**b**) DECT empirical model, and (**c**) MRI and sDECT-based (Model D). The specific locations indicated with green, yellow, and red bars in Fig. 5 correspond to the same locations in this figure. The comparison results for these locations are shown in Fig. 7a–c.

**Figure 7:**
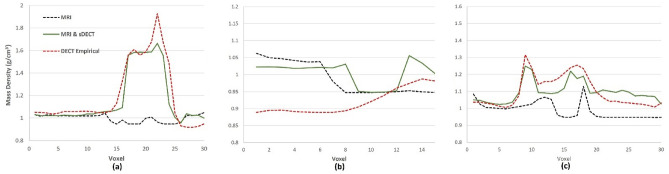
Mass density line profile (**a**–**c**) show the red, green, and blue line in Fig. 5a–f with the pixel value shown in Fig. 6.

Figure 5 illustrates the patient coronal images from T1 and T2 MRIs, DECT and sDECT HighE and LowE protocols. As shown in Fig. 4 and Table 5, in the patient application phase Model C contains only T1 and T2 MRIs, as shown in Fig. 5a,b. Model D has sDECT images as extra input, as shown in Fig. 5e–f. Figure 6a–c illustrates the mass density maps of the patient generated using different models and Fig. 7a–c illustrates the mass density line profile from the red, blue, and green line shown in Fig. 5a–f. Figure 7a illustrates the mass density line profile around the skull. At voxel 22, Model C estimated a mass density of 1.04 g/cm^3^, the DECT empirical model estimated a mass density of 1.93 g/cm^3^, while the estimation of Model D is 1.66 g/cm^3^. From voxel 1 to voxel 10, the average mass density of soft tissue predicted by Model C, Model D, and the DECT empirical models are 1.02 g/cm^3^, 1.03 g/cm^3^, and 1.05 g/cm^3^, respectively. Figure 7b portrays the mass density estimation around muscle and adipose. From voxel 1 to 8, the average mass density predication by Model C, Model D, and DECT empirical are 1.04 g/cm^3^, 1.02 g/cm^3^, and 0.88 g/cm^3^, respectively. Figure 7c illustrates the mass density profile around the clavicle bone. In the bone marrow region of voxel 12 to 14, mass density predicted by Model C, Model D, and the DECT empirical model were 1.01 g/cm^3^, 1.08 g/cm^3^, and 1.18 g/cm^3^, respectively.

**Figure 8:**
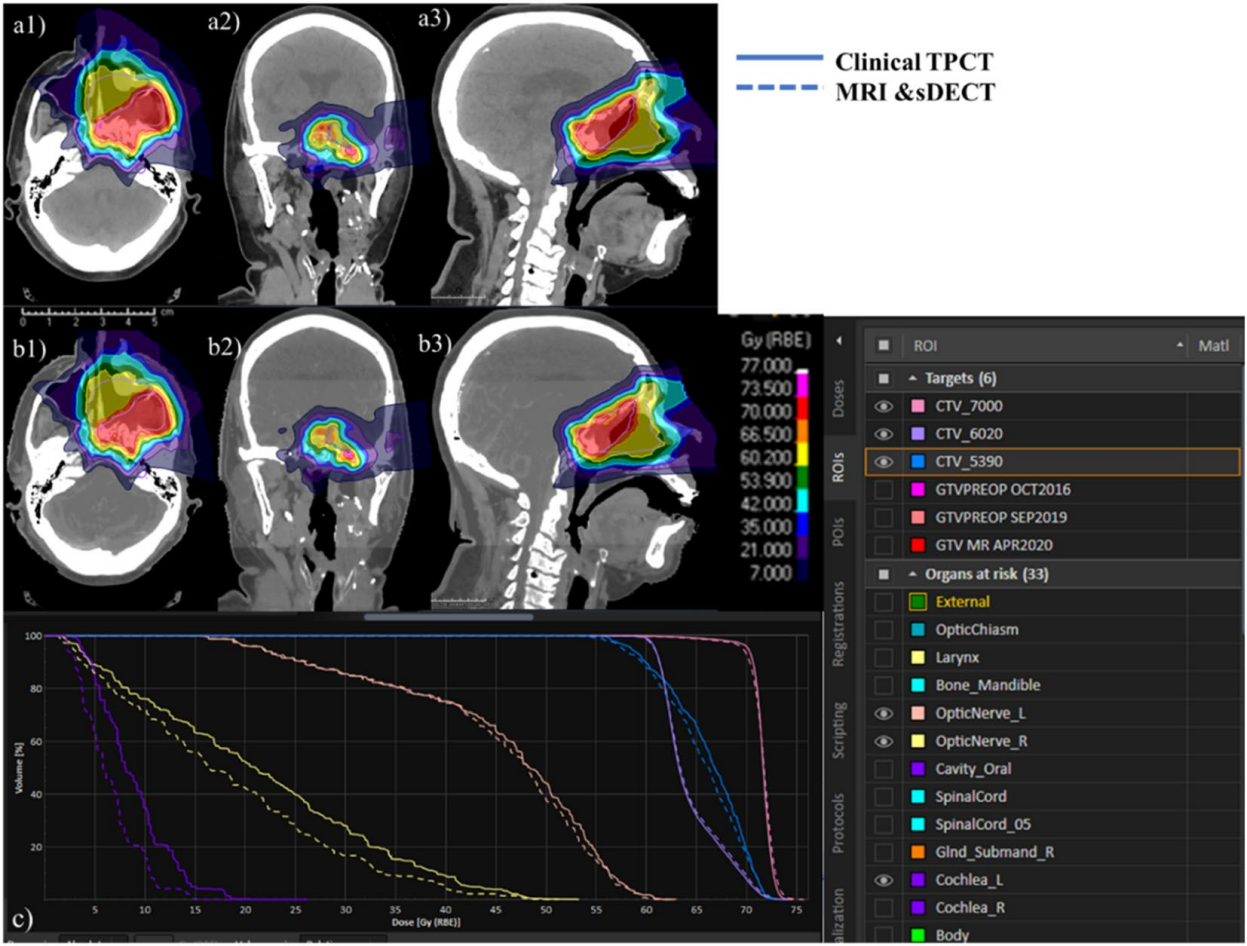
Comparison of dose distribution and DVH between treatment plan using clinical TPCT and MRI and sDECT model (Model D), (**a1**–**a3**) shows the dose distribution of treatment plan using clinical TPCT in axial, coronal and sagittal views, (**b1**–**b3**) shows the dose distribution of treatment plan using MRI and sDECT method (Model D), (**c**) shows the cumulative DVH where the solid line are the clinical TPCT based and dashed line are the MRI and sDECT (Model D) based.

Figure 8 presents a comparison of dose distribution differences for the Clinical Target Volume (CTV) and Organs at Risk (OAR) between treatment plans based on clinical TPCT and the MRI & sDECT (Model D) method. Model D employs our proposed method to generate a mass density map, which is converted into a synthetic patient CT using an institution-calibrated stoichiometric curve. This synthetic CT is then imported into RayStation for dosimetry calculations. For all three CTVs—CTV_7000, CTV_6020, and CTV_5390 the dose differences are within 1% across all volumes. For other OARs, the dose differences are considered reasonable: the left optic nerve (OpticNerve_L) shows a difference within 1% across all dose volumes; the right optic nerve (OpticNerve_R) has a difference of up to 15% at D_99_; and the left cochlea (Cochlea_L) exhibits a difference of 35% at D_98_.

## Discussion

MRI-only treatment planning, a complex yet potentially rewarding challenge within medical physics, holds great promise for enhancing patient care. Our innovative MRI-based deep learning (DL) framework is designed to match, if not surpass, traditional methods in accurately estimating mass density and Relative Stopping Power (RSP) for bone-like tissue substitute phantoms, namely Spongiosa and 45% hydroxyapatite (HA) bone. This approach is groundbreaking, as it directly correlates MRI pixel values with the mass density and RSP at specific pixels, utilizing custom-designed MRI phantoms. This technique marks a departure from conventional methods that typically involve generating synthetic CT images for treatment planning. By doing so, our framework potentially minimizes the errors associated with synthetic CT generation and the subsequent MRI-to-synthetic CT registration process. Moreover, our method has shown increased accuracy in soft tissue phantoms, highlighting its potential to outperform traditional approaches in this area. In the present work, we aim to propose a method that can convert MRI to material mass density and RSP directly. Due to the criteria of MR image formation, customized phantoms needed to be used in the experiment. However, our seven calibration tissue phantoms cover a range of material mass density focusing on the soft tissue regime which are typically used for proton TPS^51^. There are no lung and cortical bone tissue involved in this work, since the MR equivalent materials for those are quite challenging. In the proposed methodology, two DL models were trained for the phantom study: Model A contained traditional T1 and T2 MRIs as input and Model B added MRI ZTE images as input. Model B demonstrated the advantage provided by ZTE MRI over conventional MRI for differentiating bone and soft tissue. In the patient application study, two models were trained with MRI and MRI-sDECT images. The MRI-sDECT model showed improvement over the conventional DECT empirical method for estimation of patient mass density.

There has been rising interest in MRI-only treatment planning in recent years. Among those methods, the deep learning based synthetic CT (sCT) method is promising due to its superior performance^37,52^. For this approach, the workflow contains two steps, firstly the sCT images generated from MRI with DL method, and then applying SECT stoichiometric method to build CT-mass density or CT-RSP conversion. The error of this approach comes from both accuracy of sCT images and the method used for calibration. Research has been performed with advanced DL models to improve the accuracy of sCT images based on MRI^23,52,53^. To overcome the limitation of the SECT stoichiometric method, DECT was incorporated into treatment planning, and research has shown an improvement in mass density and RSP estimation^54–56^. Considering the advantage of DECT, sDECT generated from MR images with DL method for treatment planning was presented^37^. In this presented methodology, we built DL models to estimate mass density and RSP based on MRI directly and created an MRI-to-mass density and MRI-to-RSP conversion, which can improve the accuracy of conventional MRI-only treatment planning (sCT, sDECT method), since this methodology bypasses the two steps that can introduce errors (error introduced by sCT). We did not perform a comparison between our proposed method and the sDECT method because sDECT of the tissue phantoms cannot be generated. Training sDECT models require a dataset with ground truth, such as patient’s MRI and DECT images, but there is no such dataset for those homemade MRI tissue phantoms. As shown in Tables 6 and 7, Model A has improved the accuracy based on T1 and T2 MRIs, which could lead to error reduction in proton treatment planning. MRI is a multi-parametric imaging modality that not only can provide anatomical information with superior soft tissue contrast compared with CT, but also valuable functional information such as diffusion or perfusion^17^. ZTE MRI has gained more attention recently because of its superior performance for delineating bone compared with conventional T1 and T2 MRI^57–59^. In this study, Model B demonstrated better performance in estimations of mass density and RSP estimation in bone than Model A because of the additional bone signal provided by ZTE MR images.

For the patient application study, all the models gave a reasonable estimate on soft tissue mass density estimation. As shown in Fig. 7a, from voxel 1 to 10, the estimation of average mass density match the typical soft tissue mass density^5^. The T1 and T2 MRI-only model (Model C) did not perform well in bone tissue mass density estimation. As shown in Fig. 7a, Model D and the DECT empirical model gave a reasonable estimation for skull mass density, according to published research^60^. The T1 and T2 MRI-only model failed to differentiate brain and skull. We believe the main reason for this is due to the difference between patient tissue and tissue substitute phantoms, and the limitation of phantom variability (seven tissue phantoms in total with no real bone tissue). The customized MRI-visible tissue substitute phantoms are made with water, fat or other organic materials, and their T1 and T2 relaxation times are different than human tissue. For instance, the 45% HA phantom does not display the same contrast as cortical bone. The 45% HA phantom is dark on T2-STIR imaging, but unexpectedly bright on T1-Dxion imaging because of the microstructural difference between cortical bone and 45% HA phantom. Furthermore, the difference between human brain liver and pig brain liver can also contribute to the error. Another important error source comes from density and volumetric measurements, especially for the animal tissue phantoms. The MRI and sDECT model (Model D) show appropriate mass density estimation and better performance on mass density estimation than the DECT empirical model. Of note, the proposed method offers an MRI-only framework for estimating mass density. As shown in Figs. 6 and 7, this proposed model has better performance in soft tissue estimation compared with the DECT empirical model. As shown in Figs. 6 and 7b, bone marrow can be differentiated clearly because of the superior soft tissue information provided by MRI. For the bone tissue, the proposed model (Model D) can provide the appropriate mass density estimation because of the useful information provided by DL-based sDECT imaging. In the current state, the MRI and sDECT model is a reasonable choice for patient mass density estimation.

Our study has identified several limitations that need to be addressed. Firstly, DL overfitting is a common challenge that can occur when a model becomes too complex, resulting in the model memorizing the training data instead of learning the underlying patterns. However, due to the complexity of creating such phantoms, the size of our training dataset is limited, which could lead to an overfitting trained model^61,62^. Figure 8 shows the comparison of validation and training loss until 500 epochs, the validation loss is higher than training loss and not increase with more epochs, DL models with 300 epochs were used to avoid overfitting issue. To evaluate the robustness of our proposed work, we need to consider the density range of the MRI phantoms used. The density range of our MRI phantoms is from 0.94 to 1.42 g/cm^3^, which is much smaller than the conventional CT electron density phantom from 0.20 to 1.82 g/cm^3^^34^. Therefore, our smaller training dataset density range could affect the accuracy of our proposed model when dealing with very high dense materials (such as skull and implant). Secondly, there is no real bone tissue material in our training dataset. Although MRI has high soft tissue contrast, MR imaging of hard tissues such as bone and teeth is still challenging because of the low proton content^63^. Our bone tissue phantom, containing 45% HA, was designed to mimic cortical bone in density. However, since it contains 55% water and has a very high MRI signal, it is significantly different from typical bone tissue, which may reduce the performance of our MRI-only model for patient mass density estimation. Thirdly, the accuracy of the deep learning (DL) networks in our study is currently constrained due to the nature of the phantoms used for training. These phantoms are constructed using materials such as water, fat, or animal tissue, which have T1 and T2 relaxation times that differ from those of human tissues. Consequently, this discrepancy impacts the precision of our method in generating patient-specific Relative Stopping Power (RSP) maps. At this stage, the proposed method has not yet reached the level of accuracy required for generating patient RSP maps that are suitable for dosimetry calculations in a clinical setting. Therefore, further refinement and validation are needed before it can be reliably used for clinical dosimetry purposes. To address these limitations, we have incorporated synthetic CT techniques in our patient application study. This is a realistic choice we have made for the current stage, given the limitations of our current training dataset. However, if we create more MRI phantoms that cover a wide density range and include bone tissue phantoms in the future, our proposed MRI-only model could become a promising method for patient mass density estimation. A dosimetry calculation was conducted to evaluate the dose distribution differences between clinical TPCT and our proposed method (Model D). Model D demonstrated a close match with the treatment plan based on clinical TPCT. Additionally, for OARs, the differences between the proposed method and the clinical plan were reasonable, highlighting the potential of Model D for clinical application.

**Figure 9:**
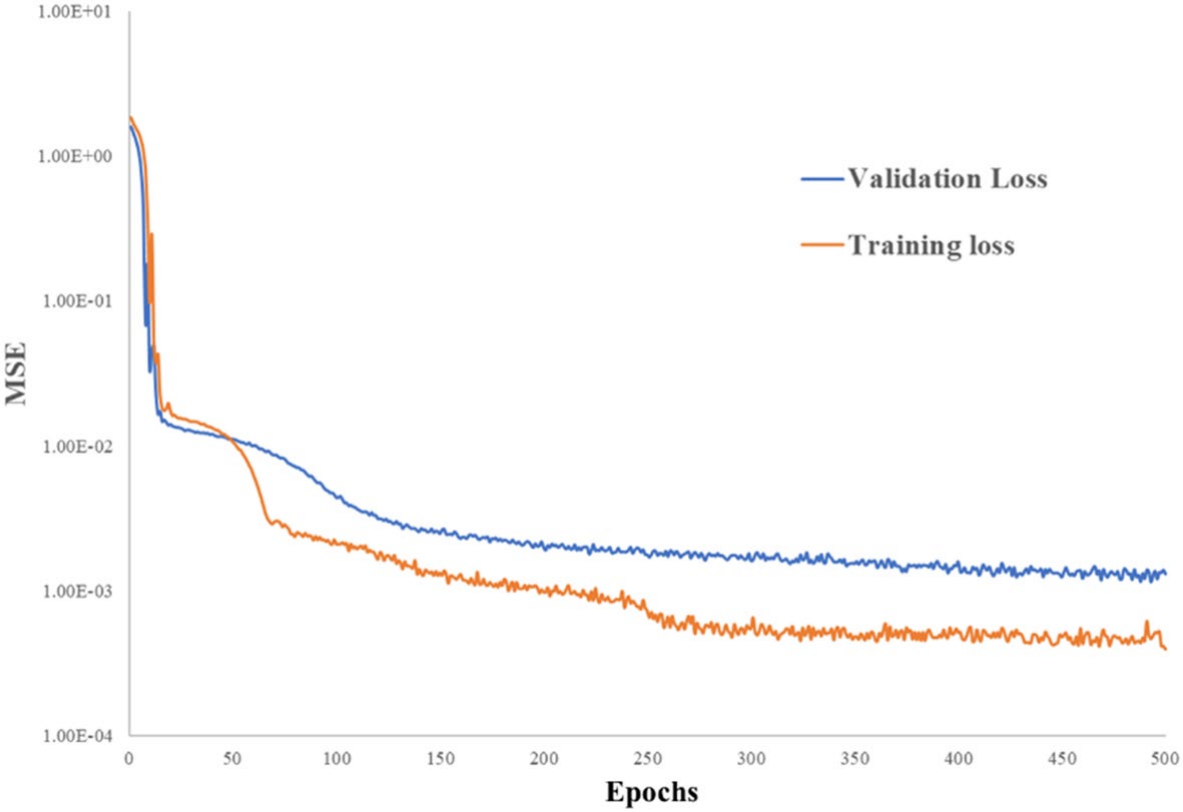
FCNN loss (total loss) curves comparison (model A).

Current clinical proton MC treatment planning system (TPS)^51^ still requires a CT-number-to-mass-density curve to characterize tissues from CT images based on five fundamental human tissues compositions including lung, adipose, muscle, cartilage, and bone^5^. The TPS usually includes a pre-tabulated material composition library^64^, which can be queried by material mass density. The current clinical robust proton treatment planning usually reserves a 2.5% range uncertainty margin due to CT-based material characterization^65^. Thus, accurate material mass density mapping will lead to a good approximation for tissue compositions. The focus of this work is to investigate the feasibility of an MRI-only material characterization method for accurate material density mapping. Seven MRI visible phantoms were designed to verify the proposed method. Manufacturing of such MRI visible phantoms is challenging due to the fundamental of MRI imaging physics, which requires the phantoms to include water or fat. The phantoms also need to conserve the effective atomic numbers, electron density, and mass density. In the future, additional customized MRI-visible phantoms will be essential for extending the training dataset, and the incorporation of ZTE MRI with experimental measurement^66^ into clinics can potentially improve the robustness of DL MRI-only models (Model C).To enhance the robustness and applicability of our study, we will develop more home-made MRI phantoms that represent a wider range of human tissues (especially bone- like and lung-like tissue phantoms). This will enable us to expand our datasets and improve the generalizability of our findings. Additionally, dosimetry calculations will serve as the endpoint of our research. By incorporating these calculations, we aim to provide a more comprehensive understanding of the relationship between MRI and material mass density. To enhance the performance of the proposed method, it is possible to integrate more advanced deep learning models into the framework^67^. Future investigation should explore the uncertainty while using different MRI sequences for material characterization. This information will be vital for improving the accuracy and precision of treatment planning in radiotherapy (Fig. 9).

## Conclusions

A DL framework was proposed to demonstrate the feasibility of estimating mass density and RSP based on MRI only. The DL model utilizing T1 and T2 MRIs demonstrated better performance than the DECT empirical model for estimating mass density and RSP. ZTE MRI can improve the DL model performance of estimating mass density and RSP in bone. The MRI and sDECT DL model showed appropriate patient bone tissue density estimation and provided superior information in soft tissue. The proposed framework has the potential to improve the quality of current MRI-only treatment planning for proton radiotherapy by providing more accurate mass density and RSP estimation. Based on the previous work^64^, the accuracy of material density maps dominates the uncertainty of current clinical MC dose calculation in the TPS.

This work is currently in a preliminary stage, and significant further efforts are required before it can be applied clinically. The future work will also focus on exploring the dosimetry impacts based on real treatment plans to investigate the feasibility of clinical application.

## Data Availability

The datasets generated during and/or analyzed during the current study are available from the corresponding author on reasonable request.
