# Author Correction: MRI-only based material mass density and relative stopping power estimation via deep learning for proton therapy: a preliminary study

**DOI:** 10.1038/s41598-024-65365-x

**Published:** 2024-06-25

**Authors:** Yuan Gao, Chih-Wei Chang, Sagar Mandava, Raanan Marants, Jessica E. Scholey, Matthew Goette, Yang Lei, Hui Mao, Jeffrey D. Bradley, Tian Liu, Jun Zhou, Atchar Sudhyadhom, Xiaofeng Yang

**Affiliations:** 1 Department of Radiation Oncology and Winship Cancer Institute, Emory University, Atlanta, GA 30308 USA; 2 GE Healthcare, Atlanta, GA 30308 USA; 3 Department of Radiation Oncology, Dana-Farber Cancer Institute, Brigham and Women’s Hospital, and Harvard Medical School, Boston, Massachusetts USA; 4 Department of Radiation Oncology, The University of California, San Francisco, CA 94143 USA; 5 Department of Radiology and Imaging Sciences and Winship Cancer Institute, Emory University, Atlanta, GA USA; 6 Radiation Oncology, Mount Sinai Medical Center, New York, NY USA

Correction to: *Scientific Reports* 10.1038/s41598-024-61869-8, published online 15 May 2024

In the original version of this Article Atchar Sudhyadhom was incorrectly affiliated with ‘GE Healthcare, Atlanta, GA, 30308, USA’. The correct affiliation is listed below.

Department of Radiation Oncology, Dana-Farber Cancer Institute, Brigham and Women’s Hospital, and Harvard Medical School, Boston, Massachusetts, USA.

The original Article has been corrected.

